# Supplementary material for: Potential Rapid Diagnostics, Vaccine and Therapeutics for 2019 Novel Coronavirus (2019-nCoV): A Systematic Review
Source: J Clin Med. 2020 Feb 26;9(3):623. doi: 10.3390/jcm9030623 (PMC7141113; doi:10.3390/jcm9030623)
Supplement: Supplementary file 1 [file jcm-09-00623-s001.zip › jcm-731887-SI.docx]

Supplementary Material

Table S1. Example of full search strategy in Pubmed

| Outcome | Search Terms |
| --- | --- |
| Diagnosis (2019-nCoV) | ((((rapid diagnostic test) OR diagnos*) OR point of care kit)) AND ((((novel coronavirus) OR wuhan virus) OR 2019-nCoV)) Filters: Publication date from 2019/12/01 to 2020/02/06 |
| Therapeutic drugs (2019-nCoV) | (novel coronavirus) AND (drugs OR therapeutics) Filters: Publication date from 2019/12/01 to 2020/12/31 |
| Vaccines (2019-nCoV) | (vaccine) AND ((((novel coronavirus) OR wuhan virus) OR 2019-nCoV)) Filters: Publication date from 2019/12/01 to 2020/02/06 |
| Diagnosis (SARS and MERS) | (((rapid diagnostic test) OR point of care kit)) AND (((((coronavirus) OR mers) OR middle east respiratory syndrome) OR sars) OR severe acute respiratory syndrome) |
| Therapeutic drugs (SARS and MERS) | ('middle east respiratory syndrome'/exp OR 'middle east respiratory syndrome' OR (middle AND east AND respiratory AND ('syndrome'/exp OR syndrome)) OR 'severe acute respiratory syndrome'/exp OR 'severe acute respiratory syndrome' OR (severe AND acute AND respiratory AND ('syndrome'/exp OR syndrome))) AND (drugs OR therapeutics) AND ([cochrane review]/lim OR [systematic review]/lim OR [meta analysis]/lim OR [controlled clinical trial]/lim OR [randomized controlled trial]/lim) |
| Vaccines (SARS and MERS) | ((vaccine) AND (((((coronavirus) OR MERS) OR middle east respiratory syndrome) OR SARS) OR severe acute respiratory syndrome)) Filters: Systematic Reviews; Clinical Trial |

Table S2: **Google Search: 2019-nCoV diagnostics**

***Selected webpage results from first two pages:***

Official Information and Guidelines from Government/International Bodies

https://[www.who.int/health-topics/coronavirus/laboratory-diagnostics-for-novel-coronavirus](http://www.who.int/health-topics/coronavirus/laboratory-diagnostics-for-novel-coronavirus)

https://[www.who.int/docs/default-source/coronaviruse/20200114-interim-laboratory-guidance-](http://www.who.int/docs/default-source/coronaviruse/20200114-interim-laboratory-guidance-) version.pdf?sfvrsn=6967c39b_4&download=true

https://[www.cdc.gov/coronavirus/2019-nCoV/clinical-criteria.html](http://www.cdc.gov/coronavirus/2019-nCoV/clinical-criteria.html)

https://[www.ecdc.europa.eu/en/publications-data/laboratory-testing-suspect-cases-2019-ncov-](http://www.ecdc.europa.eu/en/publications-data/laboratory-testing-suspect-cases-2019-ncov-) using-rt-pcr

https://[www.fda.gov/emergency-preparedness-and-response/mcm-issues/novel-coronavirus-2019-](http://www.fda.gov/emergency-preparedness-and-response/mcm-issues/novel-coronavirus-2019-) ncov

***Diagnostic Protocol and Scientific Commentaries*** https://[www.eurosurveillance.org/content/10.2807/1560-7917.ES.2020.25.3.2000045](http://www.eurosurveillance.org/content/10.2807/1560-7917.ES.2020.25.3.2000045) https://[www.elsevier.com/connect/coronavirus-information-center](http://www.elsevier.com/connect/coronavirus-information-center) https://[www.mdpi.com/1999-4915/12/2/135/pdf](http://www.mdpi.com/1999-4915/12/2/135/pdf)

***Market News and Press Releases***

https://[www.bioanalysis-zone.com/2020/01/17/researchers-develop-first-diagnostic-assay-test-](http://www.bioanalysis-zone.com/2020/01/17/researchers-develop-first-diagnostic-assay-test-) suspected-cases-novel-coronavirus/

https://[www.genengnews.com/news/coronavirus-detection-test-in-the-works-for-wuhan/](http://www.genengnews.com/news/coronavirus-detection-test-in-the-works-for-wuhan/)

https://[www.bioworld.com/articles/432651-co-diagnostics-completes-critical-step-in-developing-](http://www.bioworld.com/articles/432651-co-diagnostics-completes-critical-step-in-developing-) coronavirus-diagnostic?v=preview

https://[www.marketwatch.com/story/the-latest-coronavirus-stock-screamers-inovio-](http://www.marketwatch.com/story/the-latest-coronavirus-stock-screamers-inovio-) pharmaceuticals-co-diagnostics-2020-01-23

<https://altona-diagtics.com/en/news/assay-for-novel-coronavirus-under-development.html>

Table S3: Summary of available diagnostic platforms for 2019-nCoV

| **Type** | **Organisation** | **Date** | **Test** | **Sensitivity** | **Specificity** | **Availability** | **Turnaround** | **Costs** |
| --- | --- | --- | --- | --- | --- | --- | --- | --- |
| rRT-PCR | Charité Virology, Berlin, Germany[116,117] | 17 Jan 2020 | **Primer and Probe**  First line screening assay: E gene assay  Confirmatory assay: RdRp gene assay | First line screening assay  **Technical LOD:** 5.2 RNA copies/reaction, at 95% hit  rate  **95% CI:** 3.7-9.6 RNA copies/reaction.  Confirmatory assay  **Technical LOD:** 3.8 RNA copies/reaction, at 95% hit rate  **95% CI:** 2.7-7.6 RNA copies/reaction.  (Preliminary experiment compared single probe assay for SARS-CoV  with single probe assay for SARS-CoV-2.) | Chemical stability  No positive signal detected for non-specific reactivity of oligonucleotides.  Cross-reactivity with other coronaviruses  No reactivity with any of three assays for five coronaviruses: (HCoV) -229E, -NL63, -OC43,  -HKU1, and MERS-CoV  Tests of human clinical samples previously tested to contain respiratory viruses  All tests returned negative results for all 75 samples. | Y  • SARS-CoV genomic RNA as positive control.  • Synthetic control  RNA for SARS-CoV-2 E gene assay is available via EVAg.  • Synthetic control for SARS-CoV-2 RdRp is expected to be available via EVAg from Jan 21st onward. | 47 min 15 sec of cycle time (plus probe) for each assay | (no info) |
| rRT-PCR | School of Public Health, The University of Hong Kong (HKU)[118,119] | 16 Jan 2020 | **Primer and Probe**  Assay 1 (Target: ORF1b-nsp14)  Assay 2 (Target: N) | Positive control using SARS-CoV  RNA  Wide dynamic range of 2^-4^ to 2000  TCID^50^/reaction. | Exclusivity  Negative results against all of these preparations:  • RNA extracted from cultured viruses  • RNA from retrospective human clinical specimens previously tested positive for other infections  • RNA from control human clinical specimens | Y  • Positive control (Available from HKU)  Primers and probes:  • HKU-ORF1b-nsp14F  • HKU- ORF1b-nsp14R  • HKU-ORF1b-nsp141P  • HKU-NF  • HKU-NR  • HKU-NP | 28 min 40 sec of cycle time for each assay | (no info) |
| rRT-PCR | Chinese Center for Disease Control and Prevention[120] | 21 Jan 2020 | **Primer and Probe**  Target 1 (ORF1ab)  Target 2 (N) | (no info) | (no info) | Y | (no info) | (no info) |
| RT-PCR | Department of Medical Sciences, Ministry of Public Health, Thailand[121] | Jan 2020 | **With gel electrophoresis** | (no info) | (no info) | Y  Primers:  • NbatCoV_F1  • NbatCoV_R1 | 107 min of cycle time | (no info) |
| RT-PCR | National Institute of Infectious Diseases, Japan[122] | 23 Jan 2020 | **With gel electrophoresis** (Nested RT-PCR)  **Primer and Probe** (Real-time RT-PCR) | (no info) | (no info) | Y  Primers and probes:  • NIID_2019-nCOV_N_F2  • NIID_2019-nCOV_N_R2  • NIID_2019-nCOV_N_P2 | 81 min for Nested RT-PCR  95 min for Real-time RT-PCR | (no info) |
| rRT-PCR | Centers for Disease Control and Prevention, USA[123,124] | 24 Jan 2020 | **Primer and Probe**  3 N targets | (no info) | (no info) | Y  Primers and probes:  • 2019-nCOV_N1_F  • 2019-nCOV_N1_R  • 2019-nCOV_N1_P  • 2019-nCOV_N2_F  • 2019-nCOV_N2_R  • 2019-nCOV_N2_P  • 2019-nCOV_N3_F  • 2019-nCOV_N3_R  • 2019-nCOV_N3_P | 43 min 45 sec of cycle time for each assay | (no info) |

Table S4. Details on ongoing clinical trials on MERS-CoV vaccine

| Disease | Organisation; Candidate | Country of study; Trial Type; Study design; Study details | Inclusion criteria; outcome assessed | Study status | Reference |
| --- | --- | --- | --- | --- | --- |
| MERS | University of Oxford; ChAdOx1 MERS | United Kingdom, Oxford;  Phase I clinical trial;  Non-randomised, open-label, dose-escalation and parallel trial;  Subjects will be divided into 5 groups and receive the ChAdOx1 MERS vaccine intramuscularly, each with a different vaccination schedule.   \| Group \| # subjects \| Dose received / vaccine schedule \| \| --- \| --- \| --- \| \| 1 \| 6 \| 5 x 10^9^ vp / single dose on day of visit \| \| 2 \| 9 \| 2.5 x 10^10^ vp / single dose on day of visit \| \| 3 \| 9 \| 5 x 10^10^ vp / single dose on day of visit \| \| 4 \| 6 – 12 \| 2.5 x 10^10^ vp / first dose at week 0  2.5 x 10^10^ vp /booster at week 26 \| \| 5 \| 6 – 12 \| 2.5 x 10^10^ vp / first dose at week 0  2.5 x 10^10^ vp /booster at week 26 \| | 48 healthy adults between 18 and 50 years old, without prior receipt of an investigations product or vaccine likely to impact the trial date, history of exposure to MERS-CoV.  *Primary outcomes*   - Number of Participants with solicited and unsolicited   - Local Adverse Events   - Systemic Adverse Events   - Serious Adverse Events   for 28 days following vaccination   - Change from baseline for safety laboratory measures for 28 days following vaccination   *Secondary outcome*   - Measures of immunogenicity – quantify   - antibodies to MERS Spike protein antigen ex vivo   - ELISpot responses to MERS Spike protein antigen   to the ChAdOx1 MERS vaccine with ELISA for 12 months following completion of vaccination. | Recruiting  Study start date: March 14, 2018  Estimated completion date: July, 2021 | [125] |
| MERS | GeneOne Life Science and Inovio Pharmaceuticals; GLS-5300 | South Korea;  Phase I/IIa clinical trial;  Open-label, dose-ranging and parallel study;  GLS-5300 administered intradermally (ID) followed by electroporation at 0.3 and 0.6 mg/dose assessing 2 and 3-dose regimens in 4 separate intervention arms. | 60 healthy adults of both genders, between 19 and 70 years old, has no clinically significant acute health problems as determined from medical history and not vaccinated with any vaccine in the 4 weeks before the first dose  *Primary outcomes*   - From Day 0 to 60 weeks:   - Incidence of Adverse Events   - Frequency of injection site reactions   - Changes in safety laboratory parameters in complete blood count and liver panel tests - Injection site pain at time of vaccine administration   *Secondary outcome*   - From Day 0 to week 60   - Assessment of antigen-specific cellular immune response   - Neutralizing antibody levels   - Binding antibody levels for a 2- and 3-dose vaccine regime | Active but not recruiting  Study start date: August 28, 2018  Estimated completion date: November, 2020 | [126] |
| MERS | Gamaleya Research Institute of Epidemiology and Microbiology; BVRS-GamVac-Combi | Russia;  Phase I clinical trial;  Randomised, double-blind, parallel and placebo controlled trial (2 Phases);  Phase 1: it is planned to study   - Group 1: the safety of component 1 (40 subjects) - Group 2: the safety of prime-boost vaccination with component 1 and component 2 with an interval of 21 days in half and full dose (40 subjects)   Phase 2: it is planned to study   - the safety and immunogenicity of the vaccine as part of a placebo-controlled randomized trial (Total: 188 subjects, Intervention: 138 subjects, Placebo: 50 subjects) - Data from 20 subjects in Phase I who received the drug in selected dose will be included in the analysis of safety and immunogenicity of the second phase. | 268 healthy adults of both genders, between 18 and 55 years old, without any vaccination in the past 30 days, and acute infectious and non-infectious diseases, exacerbations of chronic diseases within 4 weeks prior to screening (Phase 1: 80 subjects, Phase 2: 188 subjects)  *Primary outcomes*   - Number of Participants with   - Adverse Events   - Serious Adverse Events   - Solicited Local Adverse Events   - Systemic Adverse Events   through the whole study (average 180 days)   - Antibody levels against the MERS-CoV glycoprotein S, measured by an enzyme-linked immunosorbent assay (ELISA) at days 0, 7, 14, 21, 28, 35 (excluding group 1 phase 1), 42, 56 in all groups in Phases 1 and 2   *Secondary outcome*   - Assessment of antigen-specific cell-mediated immune response at 0, 14 and 28 days from the start of vaccination compared to baseline values (phase 1, phase 2) and placebo (phase 2) - Neutralizing antibody levels at days 0, 14 and 28 from the start of vaccination compared to baseline values | Recruiting subjects  Study start date: November 6, 2019  Estimated completion date: December 31, 2020 | [16] |
| MERS | Gamaleya Research Institute of Epidemiology and Microbiology; BVRS-GamVac | Russia;  Phase I clinical trial;  Randomised, double-blind, parallel and placebo controlled trial (2 Phases);  Phase 1: it is planned to study the safety of a half dose compared to a full dose of vaccine (40 subjects)  Phase 2: it is planned to study  the safety and immunogenicity of the vaccine as part of a placebo-controlled randomized trial (Total: 122 subjects, Intervention: 88 subjects, Placebo: 34 subjects) | 162 healthy adults of both genders, between 18 and 55 years old, without any vaccination in the past 30 days, and acute infectious and non-infectious diseases, exacerbations of chronic diseases within 4 weeks prior to screening (Phase 1: 40 subjects, Phase 2: 122 subjects)  *Primary outcomes*   - Number of Participants with   - Adverse Events   - Serious Adverse Events   - Solicited Local Adverse Events   - Systemic Adverse Events   through the whole study (average 180 days)   - Antibody levels against the MERS-CoV glycoprotein S, measured by an enzyme-linked immunosorbent assay (ELISA) at days 0, 7, 14, 21, 28, 42, 56 in all groups in Phases 1 and 2   *Secondary outcome*   - Assessment of antigen-specific cell-mediated immune response at days 0 and[17] 14 from the start of vaccination compared to baseline values (phase 1, phase 2) and placebo (phase 2) - Neutralizing antibody levels at days 0 and 14 from the start of vaccination compared to baseline values | Recruiting subjects  Study start date: November 7, 2019  Estimated completion date: December 31, 2020 | [17] |
| MERS | University of Oxford; ChAdOx1 MERS | Saudi Arabia, Riyadh;  Phase Ib clinical trial;  Non-randomised, open-label, dose-escalation and parallel trial;  Subjects will be split into 3 study groups and receive a single dose of the ChAdOx1 MERS vaccine intramuscularly on date of visit.   \| Group \| # subjects \| Dose received \| \| --- \| --- \| --- \| \| 1 \| 6 \| 5 x 109 vp \| \| 2 \| 9 \| 2.5 x 1010 vp \| \| 3 \| 9 \| 5 x 1010 vp \| | 24 healthy middle eastern adults between 18 and 50 years old, without prior receipt of an investigations product or vaccine likely to impact the trial date, history of exposure with camels or allergic reaction to Aminoglycoside antibiotics.  *Primary outcomes*   - Number of Participants with solicited and unsolicited   - Local Adverse Events   - Systemic Adverse Events   - Serious Adverse Events   For 28 days following vaccination   - Change from baseline for safety laboratory measures for 28 days following vaccination   *Secondary outcome*   - Measures of immunogenicity – quantify   - antibodies to MERS Spike protein antigen ex vivo   - ELISpot responses to MERS Spike protein antigen   to the ChAdOx1 MERS vaccine with ELISA for 6.5 months following completion of vaccination. | Recruiting  Estimated study start date: January 1, 2020  Estimated completion date: January 1 2021 | [127] |
| MERS | Universitätsklinikum Hamburg-Eppendorf; MVA-MERS-S_DF1 | Germany;  Phase Ib clinical trial;  Randomised, double-blind, parallel and placebo controlled trial (2 parts);  Part A: A single center open-label run-in phase of two dose levels (cohort 1 "low dose": 2x10^7^ PFU, cohort 2 "high dose": 2x10^8^ PFU) in 10 healthy subjects. 5 subjects will be allocated to each dose cohort and will receive immunizations intramuscularly on days 0 and 28.  Part B: This part is a two-center, randomized, double-blind, placebo-controlled, dose-finding study in approximately 150 healthy subjects. Subjects will be allocated to two different dose cohorts and a placebo cohort; each receiving three vaccine intramuscular injections at days 0, 28 or 56 and 336. | 160 healthy adults of both genders, between 18 and 55 years old, without any vaccination from 2 weeks prior to each trial vaccination (4 weeks for live vaccines) to 3 weeks after each trial vaccination in the past 30 days, and has no clinically significant acute health problems as determined from medical history and physical examination at screening visit (Part A: 10 subjects, Part B: 150 subjects).  *Primary outcomes*   - Frequency of adverse events associated with MVA-MERS-S_DF1 on days 1, 14, 29, 42, 56, 84, 168, 336 and 364   - Safety and reactogenicity will be assesssed by observation, questionaire and diary.   - Changes from baseline for safety laboratory measures will be monitored.   - Occurence of Serious Adverse Events will be collected throughout the entire study duration. - Frequency and severity of local injection site reactogenicity signs and symptoms on days 1, 14, 29, 42, 84 and 336.   *Secondary outcome*  Immunogenicity through magnitude of MERS-S-specific antibody responses measured with ELISA and neutralization assays on days 0, 14, 28, 42, 56, 70, 84, 168, 336, 364 (dependent on vaccination scheme). | Not yet recruiting  Estimated study start date: May, 2020  Estimated completion date: December 2021 | [18] |
